# Integrated 16S rDNA and Metabolomics Analysis Unveils Dietary Fiber-Induced Changes in Small Intestinal Microbiota and Metabolites of Pigs

**DOI:** 10.3390/vetsci12111034

**Published:** 2025-10-24

**Authors:** Haifei Wang, Lele Qi, Yeyi Xiao, Jian Jin, Ruihua Huang, Shenglong Wu, Wenbin Bao

**Affiliations:** 1 Key Laboratory for Animal Genetics, Breeding, Reproduction and Molecular Design, College of Animal Science and Technology, Yangzhou University, Yangzhou 225009, China; hyfiwang@yzu.edu.cn (H.W.); llqi1016@163.com (L.Q.); yeyixiao@126.com (Y.X.); dz120210008@yzu.edu.cn (J.J.); slwu@yzu.edu.cn (S.W.); 2 Institute of Swine Science, Nanjing Agricultural University, Nanjing 210095, China; rhhuang@njau.edu.cn

**Keywords:** bacterial genera, bile acid, intestinal health, microbial diversity, nutrients

## Abstract

**Simple Summary:**

This study investigated the effects of dietary fiber on the small intestinal microbial community and metabolic profiles. The high-fiber diet significantly altered the microbial community, increasing the abundance of five genera (*Terrisporobacter*, *Howardella*, *Romboutsia*, *Cellulosilyticum*, and *Intestinibacter*), and affected metabolic pathways related to carbohydrate and tryptophan metabolism. We also identified correlations between certain bacteria and bile acids. These results suggest that dietary fiber supports small intestinal health by modulating gut microbiota and enhancing metabolic functions, highlighting potential beneficial microbial targets for improving intestinal health in pigs.

**Abstract:**

Dietary fiber has proven beneficial for improving gastrointestinal function, its role in the small intestine has received comparatively less attention. In this study, ten Erhualian pigs (40 ± 0.86 kg) were randomly assigned to two groups: a basal diet and a 7% wheat bran-supplemented diet, respectively. Using 16S rDNA sequencing and LC-MS-based untargeted metabolomics, we observed significant shifts in small intestinal microbiota and metabolome. Five genera (*Terrisporobacter*, *Howardella*, *Romboutsia*, *Cellulosilyticum*, and *Intestinibacter*) showed a significant increase in abundance in pigs fed the high-fiber diet. In addition, 155 differential metabolites were identified between the two groups. Functional enrichment analysis revealed that these metabolites were particularly enriched in pathways of carbohydrate utilization and tryptophan catabolism. Correlation analysis revealed significant associations between specific microbial genera and bile acids including lithocholic acid and 23-Norcholic acid. These findings indicate that a high-fiber diet may promote tryptophan metabolism by genera such as *Clostridium_sensu_stricto_1*, *Terrisporobacter*, and *Romboutsia*, facilitating intestinal function and health. Overall, our findings provide new insights into the effects of dietary fiber on the microbial community and metabolome in the small intestine, and highlight the potential of certain bacterial genera as beneficial microbiota for promoting small intestinal health.

## 1. Introduction

Dietary fiber is a key component of fiber-rich anmal feed and has been demonstrated to beneficially influence gastrointestinal function. It is broadly categorized into soluble and insoluble fractions, both of which contribute to animal health through distinct physiological mechanisms [1]. Exploring the role of dietary fiber in animal nutrition contributes to improving feed efficiency and plays a vital role in enhancing gut health and productivity. The gut microbiota, which secretes a variety of metabolic enzymes, is critically involved in the degradation and utilization of dietary fiber in pigs [2]. Previous studies indicated that the inclusion of 5% corn bran in pig diets positively influences growth performance, feed conversion ratio, immune response, and microbial balance in the gut [3]. A comparative study evaluating different inclusion levels of wheat bran revealed significant breed-specific differences in growth performance, fiber digestibility, and gut microbiota between Erhualian and Large White pigs, and identified several bacterial genera associated with enhanced fiber digestion [4]. The mammalian gastrointestinal tract comprises the small and large intestines, each exhibiting unique microbial profiles across regions such as the lumen and mucosal surfaces [5]. While most recent studies mainly focused on the effect of dietary fiber on the large intestinal microbiota, its effect on the microbial composition and function in the small intestine remains less studied.

The small intestine serves as the primary site for nutrient digestion and absorption. Recent studies suggested that microbial communities in the small intestine are linked to obesity and may significantly influence host metabolic processes [6]. Metabolites derived from the small intestinal microbiota stimulate the secretion of gastrointestinal hormones such as GLP-1 and GIP, which are critically involved in appetite regulation and glucose homeostasis [7]. Although the breakdown and uptake of dietary fiber mainly depend on the fermentation activities of microbes within the large intestine, emerging evidence suggests that certain types of dietary fiber can also be fermented in the distal small intestine of both humans and pigs [8]. This highlights the potential role of upper gastrointestinal microbiota in modulating host health through interactions with dietary fiber. At present, there is limited understanding of the metabolic capacity of the swine small intestinal microbiome toward dietary fiber components and its consequent impact on the metabolome. Further research is therefore needed to explore how dietary fiber influences microbial community structure and metabolic activity in the small intestine.

16S rDNA gene sequencing is widely used to characterize the composition and relative abundance of gut microbiota. In the absence of direct evidence on microbial functions within the gastrointestinal tract, comparative analysis of 16S rDNA gene sequence across experimental samples allows for the association of microbial diversity with host phenotypes. This approach has facilitated the identification of numerous microbiota associated with human disease, obesity, intestinal disorders [9,10]. Meanwhile, metabolomics, as an important component of systems biology, plays a crucial role in elucidating the interactions between host metabolism and the gut microbiota. The gut microbiota actively participates in the regulation of metabolic balances, maintenance of internal environmental stability, and integrity of the intestinal mucosal barrier. For instance, untargeted fecal metabolomics analysis revealed microbial metabolic perturbations in neonates diagnosed with late-onset sepsis, providing insights into the functional interrelationships between microbiota-derived metabolites and disease pathogenesis [11].

In this study, we used 16S rDNA sequencing and untargeted LC-MS metabolomics to investigate changes in the gut microbiota and metabolite profiles within the small intestine of pigs fed a higher fiber diet. The aim of this study was to examine the effect of dietary fiber on the intestinal microbial community and its associations with the metabolome, and to identify key microbes and metabolites involved in fiber digestion. Our findings will provide new insight into the microbiota involved in dietary fiber metabolism and highlight potential candidate microorganisms that may enhance the fiber metabolic capacity of pigs.

## 2. Materials and Methods

### 2.1. Animals

Supplementation with 7% wheat bran has been proven the optimum dietary fiber level for Erhualian pigs [4]. To further investigate the effect of dietary fiber on small intestinal microbiota and metabolites, ten 102-day-old Erhualian fattening barrows with similar body weight (40 ± 0.86 kg) were selected and randomly assigned to two groups: a control group (fed a basal diet) and a treatment group (fed a diet in which 7% wheat bran was used to equivalently replace the basal diet). The nutrient composition of both diets was consistent with that used in our previous study [4]. Pigs had a 10-day adaption period with a basal diet, followed by a 28-day formal experiment. Throughout the experimental periods, pigs were allowed ad libitum access to water, and phenotypes (average daily feed intake and weight gain) were recorded using Osborne Testing Stations System (OTSS, Osborne Industries, Inc., Osborne, KS, USA). Animals were slaughtered as previously reported procedures [4]. Ileum tissue and its contents were snap frozen in liquid nitrogen and stored at −80 °C for subsequent analysis.

### 2.2. Haematoxylin-Eosin (H&E) Staining

The porcine ileal tissues were fixed in 4% paraformaldehyde, embedded in paraffin, and sectioned at 5 μm thick slices using a rotary microtome (Leica Microsystems, Hessen, Germany). For histological evaluation, sections were subjected to H&E staining, followed by a standardized dehydration protocol: sequential immersion in ethanol gradients (70%, 80%, 95%, and 100%), xylene clearing, and neutral resin mounting. The slides were cleared in xylene, mounted with neutral resin, air-dried at 37 °C overnight, and examined under a light microscope (Nikon, Shanghai, China) to assess ileal mucosal morphology. Villus length and crypt depth were assessed by measuring 10 intact villi and 10 associated crypts using Image J 1.8.0 software.

### 2.3. 16S rDNA Gene Amplicon Sequencing

Total microbial genomic DNA was extracted from intestinal content using the cetyl trimethyl ammonium bromide method. DNA integrity was determined by agarose gel electrophoresis, and concentration and purity were quantified using a Qubit Fluorometer (Thermo Fisher Scientific, Waltham, MA, USA). To construct sequencing library, the hypervariable V3–V4 regions of the bacterial 16S rRNA gene and the ITS1 region of the fungal ITS gene were amplified with barcoded primers. PCR mixture consisted of 15 μL of Phusion^®^ High-Fidelity PCR Master Mix, 0.2 µM of each primer, and 10 ng of template DNA. The thermal cycling conditions were as follows: 98 °C for 1 min, 30 cycles of 98 °C for 10s, 50 °C for 30s and 72 °C for 30s, and a final extension at 72 °C for 5 min. PCR products were assessed by electrophoresis on a 2% agarose gel. Amplicons were pooled in equimolar ratios, separated by electrophoresis on a 2% agarose gel, and purified using Qiagen Gel Extraction Kit (Qiagen, Hilden, Germany). Sequencing libraries were constructed using NEBNext^®^ Ultra™ IIDNA Library Prep Kit following the manufacturer’s protocols (NEB, Beijing, China). Library quality was evaluated by quantitative PCR, and 250 bp paired-end sequencing was performed on an Illumina NovaSeq platform.

### 2.4. Sequencing Data Analysis

Raw sequencing reads were demultiplexed according to their unique barcodes, after which barcode and primer sequences were trimmed. Raw tags were obtained by merging the paired-end reads using FLASH software (V1.2.11) [12]. Quality filtering was performed on the raw tags using fastp, and clean tags were subsequently aligned to the database to detect and remove chimera sequences using Vsearch [13]. The obtained effective tags were processed with the DADA2 plugin within QIIME2 to generate amplicon sequence variants (ASVs), retaining those with an abundance larger than 5 [14]. Taxonomic annotation and phylogenetic reconstruction were also conducted in QIIME2. α-diversity indices were calculated to assess within-sample microbial diversity, richness, and evenness. β-diversity analysis was performed to examine compositional differences between samples and compare community structures across groups. MetaStat (http://metastats.cbcb.umd.edu/ (accessed on 20 May 2025)) was performed to identify taxa showing significant differences at various taxonomic levels. Linear discriminant analysis effect size (LEfSe) was carried out detect biomarkers with significant differential abundance [15]. Functional annotation of microbial communities was inferred using PICRUSt2 [16].

### 2.5. LC-MS/MS Analysis

Ileal sample was ground with liquid nitrogen, and the homogenate was resuspended in prechilled 80% methanol. Afterwards, the suspension was incubated on ice for 5 min and centrifuged to collect the supernatant. The supernatant was diluted with LC-MS grade water to a final methanol concentration of 53%. After centrifugation at 15,000× *g*, 4 for 20 min, the supernatant was injected into a Hypesil Gold column at a flow rate of 0.2 mL/min. Quality control samples are prepared by mixing equal volumes of extracts from all samples and are used to monitor instrument stability and data reliability. Both positive and negative polarity modes were performed for subsequent LC-MS analysis. Metabolite identification was performed using a Vanquish UHPLC system coupled with an Orbitrap Q Exactive^TM^HF-X mass spectrometer (Thermo Fisher Scientific, Waltham, USA).

### 2.6. Metabolomic Data Analysis and Metabolite Identification

Raw datasets were processed using Compound Discoverer 3.1 software for peak alignment, peak picking, and metabolite quantification. Next, peak intensities were normalized to total spectral intensity. Molecular formulas were predicted based on additive ions, molecular ion peaks, and fragment ions. Peaks were matched with the mzCloud, mzVault and MassList database (ThermoFisher Scientific, Waltham, USA) to obtain accurate qualitative and relative quantitative results. Compounds with a coefficient of variation in relative peak area > 30% in quality control samples were excluded from subsequent analysis. Metabolites were annotated using the KEGG (https://www.genome.jp/kegg/pathway.html (accessed on 15 June 2025)), HMDB (https://hmdb.ca/ (accessed on 15 June 2025)), and LIPIDMaps (https://www.lipidmaps.org/ (accessed on 15 June 2025)) databases. Multivariate statistical analyses, including principal component analysis (PCA) and partial least squares-discriminant analysis (PLS-DA), were conducted using the MetaX toolkit [17]. Differential metabolites were defined as those with a variable importance in projection (VIP) > 1, *p*-value < 0.05, and fold change >1.5 or <0.667.

### 2.7. Statistical Analysis

Statistical analyses of the microbiomic and metabolomic data are described in their respective sections. Histological data are presented as mean ± standard derivation (SD). Student’s t-test was used to compare statistical significance between the two groups. Associations between microbial taxa and metabolites were evaluated using Spearman’s rank correlation analysis. A *p*-value < 0.05 was considered statistically significant. ^*^ *p* < 0.05, ^**^ *p* < 0.01.

## 3. Results

### 3.1. Effects of Dietary Fiber Supplementation on Small Intestinal Morphology

**Figure 1:**
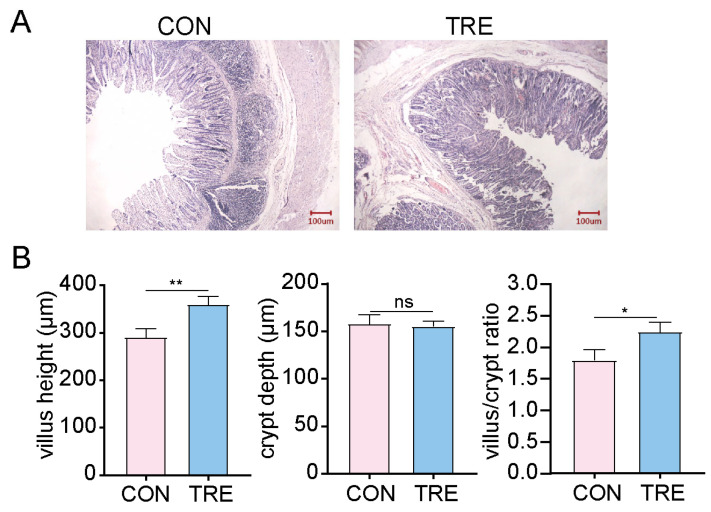
Histological analysis of the small intestine by H&E staining. (**A**) Representative H&E staining and light microscope observation of the small intestine. (**B**) Quantification of villus length, crypt depth, and the ratio of villus/crypt. Data are shown as mean ± SD (*n* = 10). CON: pigs fed a basal diet; TRE: pigs fed a 7% wheat bran-supplemented diet. Scale bar, 100 μm. * *p* < 0.05, ** *p* < 0.01, ns: not significant.

To investigate the effect of a 7% wheat bran-supplemented diet on intestinal morphology, we observed histological sections of the small intestine using HE staining (Figure 1A). The results showed that dietary supplementation with 7% wheat bran significantly increased villus height and the ratio of villus height to crypt depth compared to the control group (Figure 1B), indicating a beneficial effect of wheat bran on the physiological function of the small intestine.

### 3.2. Effects of Dietary Fiber Supplementation on Microbial Diversity in the Pig Small Intestine

**Figure 2:**
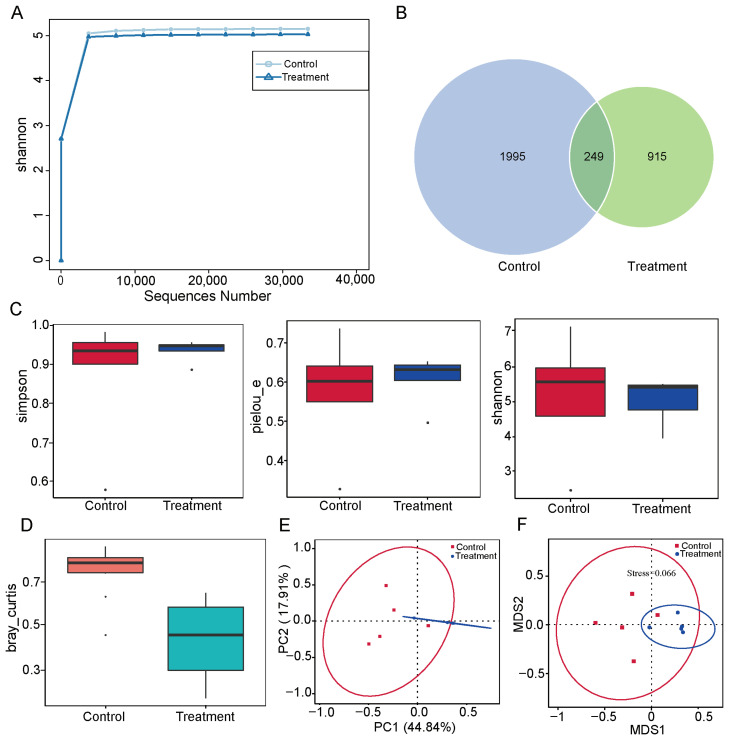
Analysis of the effects of dietary fiber supplementation on intestinal microbial diversity. (**A**) Dilution curves of the two groups. (**B**) Venn diagram of ASVs. (**C**) Test of α-diversity indices based on Simpson, Pielou’s evenness (Pielou_e), and Shannon indices. (**D**) Inter-group difference analysis of β-diversity based on Bray–Curtis. (**E**) PCoA between the two groups. (**F**) NMDS analysis based on Bray–Curtis. Control: pigs fed a basal diet; Treatment: pigs fed a 7% wheat bran-supplemented diet.

To explore the effect of dietary fiber supplementation on the microbial diversity in the small intestine, we performed 16s rDNA sequencing. Quality analysis showed that the dilution curves of all samples tended to be flat, indicating sufficient sequencing depth and adequate coverage of microbial diversity (Figure 2A). A total of 3159 distinct amplicon sequence variants (ASVs) were identified across both groups, with 249 ASVs shared between them. The control group contained 1995 unique ASVs, while the treatment group contained 915 unique ASVs (Figure 2B). Subsequent α-diversity analysis showed no significant disparities in microbial richness or evenness between the two groups (Figure 2C). In contrast, β-diversity analysis indicated notable differences in microbial community composition (Figure 2D), suggesting distinct community structures despite similar α-diversity metrics. To further visualize structural differences, we performed principal coordinate analysis (PCoA) and non-metric multidimensional scaling (NMDS) based on evolutionary distances. PCoA showed that dietary fiber supplementation altered the overall structure of the intestinal microbiota (Figure 2E), and NMDS confirmed clear separation between the microbial communities of the two groups (Figure 2F). Taken together, these results suggest that dietary fiber supplementation led to significant changes in the microbial community structure of the pig small intestine, without markedly affecting species richness or evenness.

### 3.3. Effects of Dietary Fiber Supplementation on the Composition of Small Intestinal Microbiota

**Figure 3:**
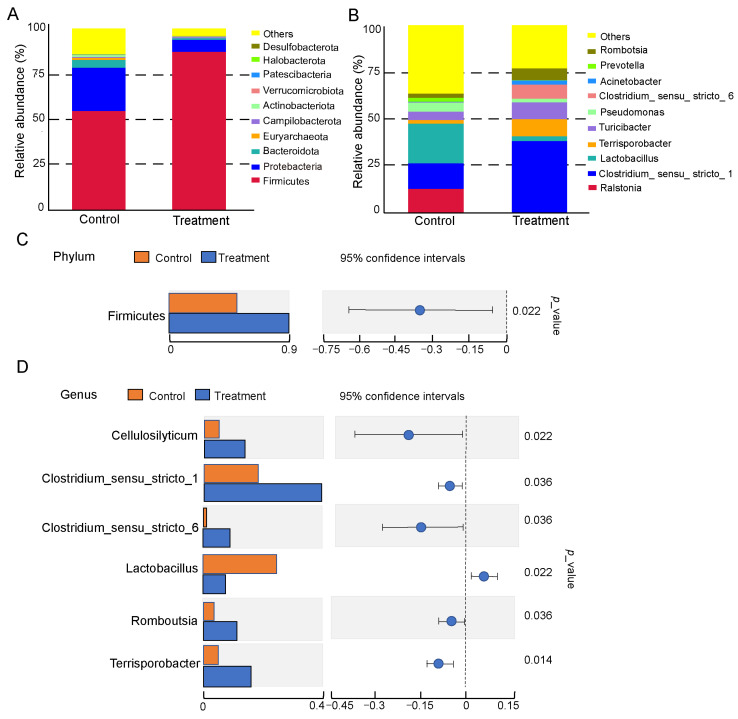
Composition of the small intestinal microbiota. (**A**) Stacked bar chart of the relative abundance of species at the Phylum level. (**B**) Stacked bar chart of the relative abundance of species at the Genus level. (**C**) Species difference analysis at the phylum level between the two groups. (**D**) Species difference analysis at the genus level between the two groups. Control: pigs fed a basal diet; Treatment: pigs fed a 7% wheat bran-supplemented diet.

To further explore the impact of dietary fiber supplementation on the small intestinal microbiota, we compared microbial profiles between the two groups. The taxonomic composition was visualized using stacked bar plots representing the top 10 most abundant phyla and genera. At the phylum level, the microbiota was mainly composed of *Firmicutes*, *Proteobacteria*, *Bacteroidetes*, and *Tenericutes*, with *Firmicutes* and *Proteobacteria* being the two most abundant phyla in both groups (Figure 3A). Overall, after feeding a diet containing 7% dietary fiber, an obvious decrease in *Proteobacteria* and an increase in *Firmicutes* were observed, indicating that dietary fiber may inhibit *Proteobacteria* while promoting the growth of *Firmicutes*. At the genus level, the dominant bacterial groups in the control group were *Roseburia*, *Clostridium*, *Lactobacillus*, and *Pseudomonas*, whereas the dominant genera in the treatment group were *Clostridium*, *Turicibacter*, *Ruminococcus*, and *Romboutsia* (Figure 3B). Differential abundance analysis showed a significant increase in *Firmicutes* at the phylum level (Figure 3C). At the genus level, significant increases were observed in *Clostridium_sensu_stricto_1*, *Terrisporobacter*, *Romboutsia*, and *Intestinibacter* in the treatment group (Figure 3D).

### 3.4. Identification of Specific Bacteria Related to Fiber Digestion

**Figure 4:**
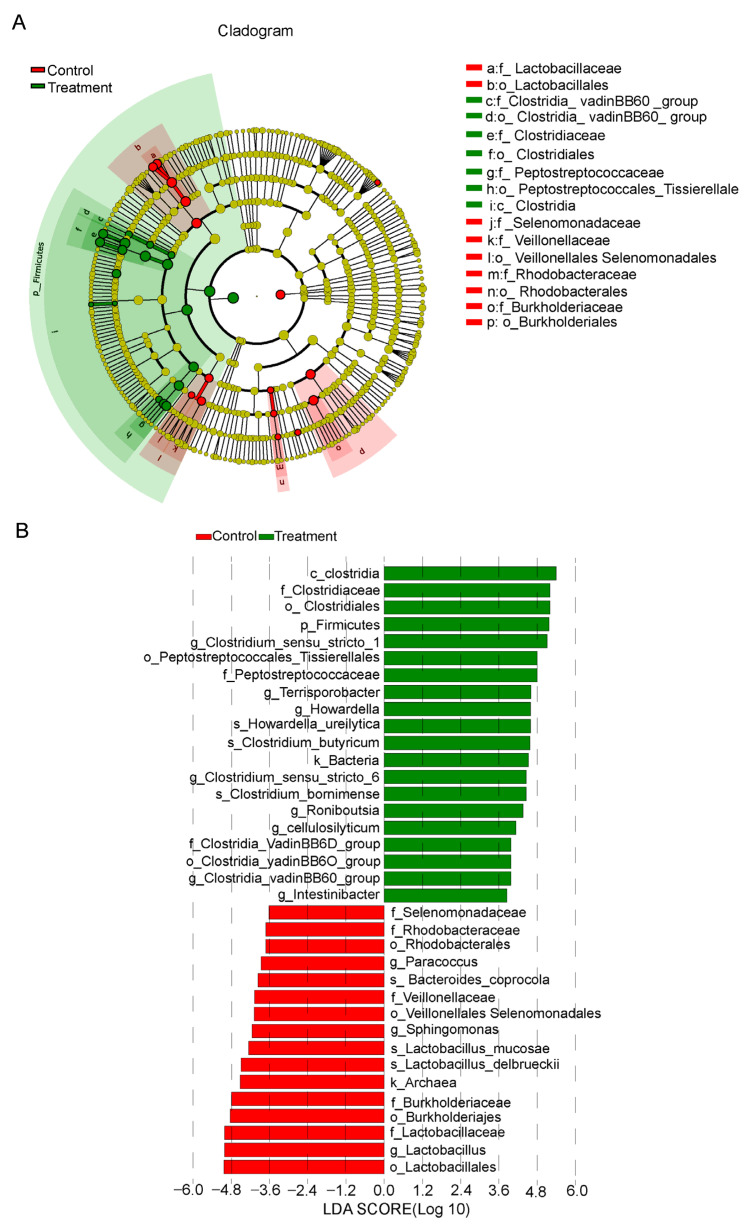
Discriminant analysis of LEfSe multilevel species differences. (**A**) Cladogram generated by LEfSe analysis. The diagram radiates from the center, representing taxonomic levels from phylum to genus. Each node represents a taxon at a specific rank, with node diameter proportional to its relative abundance. Nodes are colored according to group association: red indicates taxa enriched in the control group, and green indicates those enriched in the treatment group. (**B**) Bar chart of LDA scores. Biomarkers were defined as taxa with an LDA score. Bar color corresponds to the respective experimental group, and bar length reflects the magnitude of the effect size between groups. Control: pigs fed a basal diet; Treatment: pigs fed a 7% wheat bran-supplemented diet.

To identify bacterial taxa potentially associated with dietary fiber digestion, we conducted LEfSe analysis, generating a cladogram to visualize differentially enriched microorganisms between the two groups (Figure 4A). The control group was enriched with one domain, *Archaea*; four orders, *Lactobacillales*, *Burkholderiales*, *Veillonellales-Selenomonadales*, and *Rhodobacterales*; five families, *Lactobacillaceae*, *Burkholderiaceae*, *Veillonellaceae*, *Rhodobacteraceae*, and *Selenomonadaceae*; and three genera, *Lactobacillus*, *Sphingomonas*, and *Paracoccus*. In contrast, the treatment group exhibited significant enrichment in one domain, Bacteria; one phylum, *Firmicutes*; one class, *Clostridia*; three orders, *Clostridiales*, *Peptostreptococcales-Tissierellales*, and *Clostridia_vadinBB60_group*; three families, *Clostridiaceae*, *Peptostreptococcaceae*, and *Clostridia_vadinBB60_group*; and eight genera, *Clostridium_sensu_stricto_1*, *Terrisporobacter*, *Howardella*, *Clostridium_sensu_stricto_6*, *Romboutsia*, *Cellulosilyticum*, *Clostridia_vadinBB60_group*, and *Intestinibacter*. In the treatment group, microbial composition shifted significantly, with higher levels of *Clostridium* and *Firmicutes* (Figure 4B), while *Lactobacillus* levels significantly decreased. The genera *Terrisporobacter*, *Howardella*, *Romboutsia*, *Cellulosilyticum*, and *Intestinibacter*, which were significantly more abundant in the treatment group, may represent key biological indicators involved in dietary fiber digestion in pigs.

### 3.5. Effects of Dietary Fiber Supplementation on the Metabolite Profiles of the Small Intestine

**Figure 5:**
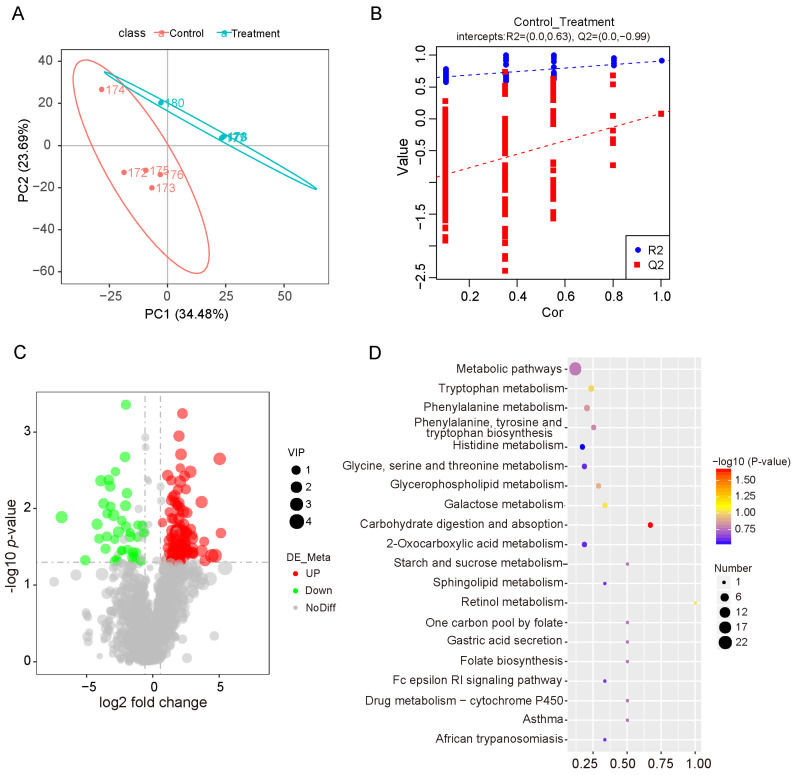
Metabolomics analysis of the small intestine. (**A**) PLS-DA score plot illustrating metabolic differences between the two groups. (**B**) Permutation test assessing the reliability of the PLS-DA model. (**C**) Volcano plot of differential metabolites between the treatment and control groups. Red and green dots represent significantly upregulated and downregulated metabolites, respectively. (**D**) Top 20 signaling pathways enriched by the differential metabolites. Control: pigs fed a basal diet; Treatment: pigs fed a 7% wheat bran-supplemented diet.

To investigate alterations in the metabolite profile, we conducted untargeted metabolome analysis using LC-MS/MS in both positive and negative ion modes. PLS-DA showed clear separation between the two groups, suggesting distinct metabolite profiles (Figure 5A). Permutation tests confirmed that the PLS-DA models were robust, accurate, and not overfitted (Figure 5B), supporting the reliability of the observed metabolic differences. Collectively, these results indicate a significant effect of dietary fiber on the intestinal metabolome. Differential metabolite analysis identified 154 differential metabolites, of which 115 were upregulated and 39 were downregulated (Figure 5C; Table S1). To elucidate the biological significance of these changes, we mapped the differential metabolites to the KEGG pathway database (Table S2). The results demonstrated that the most prominently affected metabolic pathways were related to carbohydrate digestion and absorption and tryptophan metabolism (Figure 5D).

### 3.6. Integrated Analysis of 16S rRNA Sequencing and Metabolomics Data

**Figure 6:**
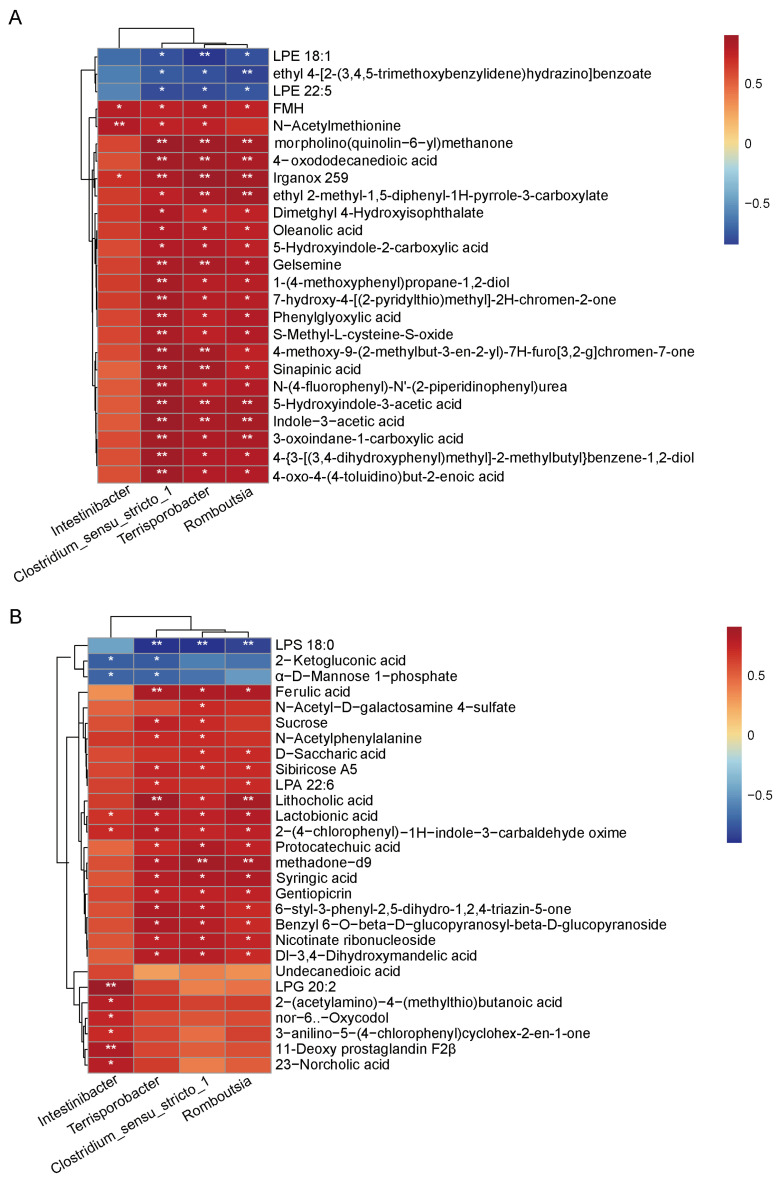
Heatmap of correlations between microbiota and differential expression metabolites in positive (**A**) and negative (**B**) ion modes. The horizontal axis represents genera with significant differences between the two groups, and the vertical axis represents the differential metabolites. Graded color represents the correlation coefficient from −1 to 1. * *p* < 0.05, ** *p* < 0.01.

Although dietary fiber is not typically absorbed in the small intestine [18], our data demonstrate obvious alterations in metabolite levels, suggesting that these differential metabolites may be a consequence of microbial community shifts. To assess microbiome-metabolite associations, we performed Spearman correlation analysis between microbiota and differential metabolites in both positive (Figure 6A) and negative (Figure 6B) ion modes, respectively (Table S3). The results revealed multiple significant correlations between intestinal microbiota and metabolites. Notably, tryptophan metabolic intermediates showed strong correlations with the abundance of specific bacterial genera. The genera *Clostridium_sensu_stricto_1*, *Terrisporobacter*, *and Romboutsia* showed significant positive correlations with a subset of metabolites. Specifically, *Clostridium_sensu_stricto_1* (ρ = 0.73, *p* = 0.031), *Romboutsia* (ρ = 0.85, *p* = 0.006), and *Terrisporobacter* (ρ = 0.88, *p* = 0.003) were significantly correlated with lithocholic acid, respectively. Additionally, *Intestinibacter* was significantly correlated with 23-Norcholic acid (ρ = 0.78, *p* = 0.013).

## 4. Discussion

The gut microbial environment is substantially modulated by dietary fiber, which contributes to improved intestinal health. As the gut microbiota plays an essential role in host physiology, numerous studies have focused on its dynamic interactions within the colon, revealing critical host-microbiota associations [19]. Similarly, the microbiota of the distal small intestine contributes importantly to digestive functions. In this study, we investigated microbial changes in the small intestine and intestinal metabolome of pigs fed a high-fiber diet using 16S rDNA sequencing and metabolomics. We observed a significant increase in villus height in the intestines of pigs receiving higher fiber, indicating an expanded surface area and enhanced nutrient absorption capacity. Previous studies have similarly reported that dietary fiber increases villus height in the jejunum and ileum of weaned piglets and stimulates the secretion various digestive enzymes [20,21]. Zhao et al. further demonstrated that appropriate dietary fiber levels improve intestinal morphology and growth performance in commercial pigs [22]. Together, these findings suggest the beneficial effects of dietary fiber on intestinal structure and function. However, it should be noted the inclusion of 7% wheat bran may introduce slight alterations in dietary nutrient composition, which could act as a confounding factor in the interpretation of the observed effects.

Emerging studies revealed that fiber digestion primarily relies on the activity of gut microbiota. In our work, no significant differences in α-diversity indices were identified, suggesting that the overall microbial richness and evenness remain largely stable under higher dietary fiber. It is known that shifts in the microbiome can occur without changes in α-diversity, often reflected in β-diversity or functional potential [23]. Our subsequent analyses revealed significant β-diversity between the two groups, indicating a reorganization of taxa rather than a change in overall diversity. In pigs, the phyla *Firmicutes* and *Bacteroidetes* typically dominate the gut microbiota [24]. Shin et al. found that *Proteobacteria* are closely associated with gut health status in humans, with lower abundances contributing to microbial balance [25]. Elevated levels of *Proteobacteria* were associated with multiple pathologies, including metabolic disorders and malignancies. In the present study, *Firmicutes* and *Bacteroidetes* dominated the intestinal microbiota in the treatment group. We found that a high-fiber diet resulted in a marked reduction in the abundance of *Proteobacteria* concomitant with an increase in *Firmicutes*. *Firmicutes* participate in maintaining host wellbeing by degrading dietary fibers and cellulose and by establishing functional interactions with the gut mucosa [26]. These findings indicate that higher dietary fiber intake may suppress *Proteobacteria* proliferation while enhancing the expansion of *Firmicutes*. However, gut microbiota composition differs across pig breeds, resulting in differences in digestive functions and microbial profiles [27]. As this study was conducted exclusively in Erhualian pigs, the applicability of these findings to commercial pigs warrants further investigations.

*Clostridium_sensu_stricto_1*, an important symbiotic bacterial genus in the gut, plays a significant role in the stability of the intestinal environment [28]. *Romboutsia* contributes to intestinal motility and the production of short-chain fatty acids (SCFAs) [29]. Qi et al. found that *Schisandra chinensis* treatment increased the abundance of *Intestinibacter* in antibiotic-associated diarrhea rats and significantly enhanced SCFAs levels in the gut. Additionally, intestinal homeostasis is modulated by microbiota-derived SCFAs [30]. Therefore, we speculate that dietary fiber supplementation increases the abundance of beneficial gut microbes, enhances SCFAs production, and further benefits intestinal health.

Our data demonstrate that a high-fiber diet caused marked alterations in the small intestinal metabolome, consistent with previous reports that fiber-rich diets significantly affect metabolite profiles in the intestinal tract [31]. Further, carbohydrate metabolism and tryptophan metabolism were significantly enriched by differential metabolites. Previous studies showed that metabolites within these pathways modulate host energy balance and support intestinal health [32]. Specifically, tryptophan and its metabolites (e.g., kynurenine, xanthurenic acid, 5-hydroxytryptophan) modulate intestinal health by enhancing epithelial proliferation, maintaining barrier integrity, regulating immune responses, and shaping microbial composition [33,34]. Herein, significant enrichment of metabolites involved in tryptophan metabolism suggests that dietary fiber may influence tryptophan metabolic processes by modulating the composition and metabolic activity of the gut microbiota. These findings indicate a potential positive impact of dietary fiber on maintaining intestinal health and function.

Dietary fiber is poorly absorbed in the small intestine, alterations in metabolite profiles in this region primarily reflect microbial activity. Correlation analysis revealed significant associations between specific microbial genera and metabolite levels. In particular, *Clostridium_sensu_stricto_1*, *Terrisporobacter*, and *Romboutsia* showed a significant positive correlation with lithocholic acid. Metabolites derived from *Clostridium_sensu_stricto_1* (e.g., butyrate, secondary bile acids, indolepropionic acid) exert probiotic effects by fueling intestinal epithelial cells, strengthening the gut barrier, and modulating immune responses [35]. Similarly, *Romboutsia* has been associated with the metabolome of bile acids, triglycerides, amino acids and their derivatives, as well as organic acids [36]. Our findings suggest that a high-fiber diet may promote tryptophan metabolism through the activity of genera such as *Clostridium_sensu_stricto_1*, *Terrisporobacter*, and *Romboutsia*. These results underscore the beneficial role of dietary fiber in shaping intestinal microbiota and metabolite profile, thereby promoting the intestinal health and function.

There are several limitations of our study. First, the relatively small sample size may limit the statistical power, potentially leading to undetected biologically relevant differences. Second, the taxonomic resolution of 16S rRNA sequencing is limited, unable to achieve precise identification at the species or strain level. Future studies could employ shotgun metagenomic sequencing or targeted functional gene analysis to achieve higher taxonomic and functional resolution. Third, although our multi-omics approach revealed correlations between microbial composition and metabolic profiles, functional validation such as targeted metabolomics and enzyme activity assays, will be required to confirm the predicted metabolic functions and establish causative links.

## 5. Conclusions

In summary, this study demonstrates that dietary fiber can obviously alter the microbial community and metabolic profile in the pig small intestine. Key metabolites such as lithocholic acid and 23-Norcholic acid were identified and correlated with specific bacterial genera, suggesting their role in enhancing intestinal barrier function and overall gut health. These findings provide novel insights into diet-microbiota-metabolite interactions and highlight potential beneficial microbial targets for improving intestinal health in pigs.

## Data Availability

The original contributions presented in this study are included in the article/Supplementary Materials. Further inquiries can be directed to the corresponding author.

## Supplementary Materials

The following supporting information can be downloaded at: https://www.mdpi.com/article/10.3390/vetsci12111034/s1, Table S1: Differential metabolites between dietry fiber-treated and control groups; Table S2: Significantly enriched pathways by differential metabolites; Table S3: Correlations between differential metabolites and bacterial genera.
